# Draft Genome Sequence of Serratia rubidaea, a Potential Opportunistic Pathogen Isolated from Food in Italy

**DOI:** 10.1128/MRA.00707-21

**Published:** 2021-07-29

**Authors:** Alexandra Chiaverini, Marco Di Domenico, Ilaria Del Matto, Franca Rossi, Gabriella Centorotola, Alessandra Cornacchia, Nicola Cornacchione, Domenico Petrone, Giorgio Iannitto, Lucio Marino, Cesare Cammà, Francesco Pomilio

**Affiliations:** a Hygiene in Food Technology and Animal Feeds, Istituto Zooprofilattico Sperimentale dell’Abruzzo e del Molise “G. Caporale,” Teramo, Italy; b National Reference Centre for Whole Genome Sequencing of Microbial Pathogens: Database and Bioinformatic Analysis, Istituto Zooprofilattico Sperimentale dell’Abruzzo e del Molise “G. Caporale,” Teramo, Italy; c Diagnostic Section, Istituto Zooprofilattico Sperimentale dell’Abruzzo e del Molise “G. Caporale,” Campobasso, Italy; d ASREM, Campobasso, Italy; University of Maryland School of Medicine

## Abstract

Serratia rubidaea has emerged in recent years as an opportunistic nosocomial pathogen. Here, we present the draft genome sequence of an isolate derived from an industrial meat food product purchased in a large-scale retail store that revealed fluoroquinolone, β-lactam, and aminoglycoside resistance genes and two different host-unspecific prophages.

## ANNOUNCEMENT

Serratia rubidaea is a Gram-negative, facultative anaerobic, rod-shaped bacterium of the class *Gammaproteobacteria*, family *Enterobacteriaceae*. It is widespread in the environment and causes sporadic severe opportunistic nosocomial infections (1–5).

In this study, we investigated a strain isolated in 2018 from chicken wurstel which had developed an atypical fuchsia pink color a few minutes after the package was opened by the consumer at home. The sample was brought to the laboratory, and 10 g was submitted for microbiological testing by suspension in 90 ml of sterile physiological solution (9 g/liter NaCl), homogenization in a Stomacher 400 blender (VWR International S.r.l., Milan, Italy), serial dilutions and plating by inclusion in cetrimide-fucidin-cephalothin agar (CFC) (Liofilchem, Roseto degli Abruzzi, Italy). A pure colony of a microorganism presenting at a high bacterial load (1.1 × 10^7^ CFU/g), appearing smooth, convex, circular, red/brown, and with a diameter of about 1.5 mm, was obtained after two streaks onto brain heart infusion (BHI) agar (Liofilchem) and incubated at 37°C for 24 h. The isolate was then identified as Serratia rubidaea using the Vitek 2 system (bioMérieux Italia S.p.a., Grassina, Florence, Italy) with the identification card for Gram-negative organisms (bioMérieux, Marcy l’Etoile, France). Aliquots of biomass from the pure colony collected from streaks on BHI agar were stored at −80°C in Microbank tubes (Biolife Italiana S.r.l., Milan, Italy). DNA was extracted according to the method by Portmann et al. (6), with minor modifications, using the QIAamp DNA minikit (Qiagen, Hilden, Germany), and quantified using the Qubit double-stranded DNA (dsDNA) high-sensitivity (HS) assay kit (Thermo Fisher Scientific, Waltham, MA).

Starting from 1 ng, a paired-end library was prepared using the Nextera XT DNA kit (Illumina, San Diego, CA) according to the manufacturer’s protocol. Whole-genome sequencing was performed on the NextSeq 500 platform (Illumina) using the NextSeq 500/550 midoutput reagent cartridge v2 (300 cycles; standard 150-bp paired-end reads). A total of 3,851,059 reads were retained from 4,031,372 raw reads after trimming using Trimmomatic v036 (7). Quality control was then performed using FastQC v0.11.5 (8). *De novo* assembly was performed using SPAdes v3.11.1 (9) and quality checked using QUAST v4.3 (10).

The final genome assembly resulted in 5,039,641 bp combined in 58 contigs, with a GC content of 57.86%. The mean coverage was 103×, the *N*_50_ value was 274,965 bp, and the *L*_50_ value was 6. Genome annotation using the NCBI Prokaryotic Genome Annotation Pipeline (PGAP) (11) returned 4,678 coding DNA sequences (CDS), 5 rRNAs, and 72 tRNAs.

Using KmerFinder (12), the predicted species was Serratia rubidaea, close to strain NCTC10848 (GenBank accession number LS483492.1).

Using ABRicate (13), fluoroquinolone, β-lactam, and aminoglycoside resistance genes were predicted using public databases such as CARD (14), ARG-ANNOT (15), and ResFinder (16). Moreover, two intact prophages with a score value of 150 were detected using PHASTER (17), one related to Escherichia phage 186 (GenBank accession number NC_001317.1) and the other to *Erwinia* sp. phage ENT90 (NC_019932.1). No plasmids were detected using PlasmidFinder (18). Default parameters were used for all the bioinformatics tools mentioned.

The draft genome sequence presented here may help us better identify the genes responsible for the intrinsic and acquired resistome of the genus *Serratia* and will help clarify the mechanisms leading to the tendency of phages to escape bacterial resistance. Furthermore, the fact that this isolate derived from an industrial meat food product purchased in a large-scale retail store suggests further investigation into food as a source of the strains responsible for human infections.

### Data availability.

This whole-genome shotgun project has been deposited in GenBank under accession numbers JAHAVC000000000 and GCA_018587965.1 and SRA accession number SRR15082308. The version described in this paper is the first version. The BioProject accession number is PRJNA728844. The BioSample accession number is SAMN19104791.
